# Postmastectomy Breast Reconstruction in Patients with Non-Metastatic Breast Cancer: An Ontario Health (Cancer Care Ontario) Clinical Practice Guideline

**DOI:** 10.3390/curroncol32060357

**Published:** 2025-06-17

**Authors:** Toni Zhong, Glenn G. Fletcher, Muriel Brackstone, Simon G. Frank, Renee Hanrahan, Vivian Miragias, Christiaan Stevens, Danny Vesprini, Alyssa Vito, Frances C. Wright

**Affiliations:** 1 Plastic and Reconstructive Surgery, University Health Network, Toronto, ON M5G 2C4, Canada; 2 Department of Surgery, University of Toronto, Toronto, ON M5T 1P5, Canada; hanrahanr@rvh.on.ca; 3 Program in Evidence-Based Care, Department of Oncology, McMaster University, Hamilton, ON L8V 5C2, Canada; gfletche@mcmaster.ca; 4 Department of Surgical Oncology, London Regional Cancer Program, London Health Sciences Centre, London, ON N6A 5W9, Canada; muriel.brackstone@lhsc.on.ca; 5 Departments of Surgery and of Oncology, University of Western Ontario, London, ON N6A 5W9, Canada; 6 Department of Surgery, The Ottawa Hospital, Ottawa, ON K1H 8L6, Canada; sfrank@toh.ca; 7 Division of Plastic and Reconstructive Surgery, University of Ottawa, Ottawa, ON K1Y 4E9, Canada; 8 Department of Surgery, Royal Victoria Regional Health Care Centre, Barrie, ON L4M 6M2, Canada; 9 Department of Surgery, McMaster University, Hamilton, ON L8S 1C7, Canada; 10 Patient Representative, Toronto, ON, Canada; 11 Radiation Treatment Program, Royal Victoria Hospital, Barrie, ON L4M 6M2, Canada; stevensc@rvh.on.ca; 12 Departments of Radiation Oncology and of Family and Community Medicine, University of Toronto, Toronto, ON M5T 1P5, Canada; 13 Department of Radiation Oncology, Odette Cancer Centre, Sunnybrook Hospital, Toronto, ON M4N 3M5, Canada; danny.vesprini@sunnybrook.ca; 14 Department of Radiation Oncology, University of Toronto, Toronto, ON M5T 1P5, Canada; 15 Patient Representative, Port Perry, ON, Canada; alyssavito87@gmail.com; 16 Department of Surgery, Sunnybrook Health Sciences Centre, Toronto, ON M4N 3M5, Canada; frances.wright@sunnybrook.ca; 17 Departments of Surgery and of Health Policy, Management and Evaluation, University of Toronto, Toronto, ON M5T 1P5, Canada; 18 Surgical Oncology Program, Ontario Health (Cancer Care Ontario), Toronto, ON M5G 2L3, Canada

**Keywords:** breast reconstruction, breast implants, autologous reconstruction, autologous fat grafting, acellular dermal matrix, prepectoral, subpectoral, immediate reconstruction, delayed reconstruction, nipple-sparing mastectomy

## Abstract

Several postmastectomy breast reconstruction techniques and procedures have been implemented, although with limited evaluation of benefits and adverse effects. We conducted a systematic review on the plane and timing of reconstruction, and on the use of nipple-sparing mastectomy, acellular dermal matrix, and autologous fat grafting as the evidence base for an updated clinical practice guideline on breast reconstruction for Ontario Health (Cancer Care Ontario). Both immediate and delayed reconstruction may be considered, with preferred timing depending on factors such as patient preferences, type of mastectomy, skin perfusion, comorbidities, pre-mastectomy breast size, and desired reconstructive breast size. Immediate reconstruction may provide greater psychological or quality of life benefits. In patients who are candidates for skin-sparing mastectomy and without clinical, radiological, and pathological indications of nipple-areolar complex involvement, nipple-sparing mastectomy is recommended provided it is technically feasible and acceptable aesthetic results can be achieved. Surgical factors including incision location are important to reduce necrosis by preserving blood supply and to minimize nerve damage. There is a role for both prepectoral and subpectoral implants; risks and benefits will vary, and decisions should be made during consultation between the patient and surgeons. In patients who are suitable candidates for implant reconstruction and have adequate mastectomy flap thickness and vascularity, prepectoral implants should be considered. Acellular dermal matrix (ADM) has led to an increased use of prepectoral reconstruction. ADM should not be used in case of poor mastectomy flap perfusion/ischemia that would otherwise be considered unsuitable for prepectoral reconstruction. Care should be taken in the selection and handling of acellular dermal matrix (ADM) to minimize risks of infection and seroma. Limited data from small studies suggest that prepectoral reconstruction without ADM may be feasible in some patients. Autologous fat grafting is recommended as a treatment for contour irregularities, rippling following implant-based reconstruction, and to improve tissue quality of the mastectomy flap after radiotherapy.

## 1. Introduction

Mastectomy without reconstruction may have negative impacts on body image, sexual health, and feelings of attractiveness, and presents a visual reminder of cancer. Reconstruction with autologous tissue or implants restores appearance and is associated with better quality of life (QoL) [1,2,3,4,5]. Patient satisfaction varies, depending on expectations, values, disease factors, and surgical expertise. While appearance after reconstruction is often satisfactory or excellent, lack of sensation is considered the norm despite advances in the field. Aesthetic flat closure may be preferred to reconstruction by some women; however, this was outside the scope of this guideline.

Reconstructive time may be immediately after mastectomy or delayed until months or years later. The optimal timing has been unclear and may be influenced by whether postmastectomy radiation therapy (PMRT) will be administered [6,7,8,9,10]. Skin-sparing mastectomy (SSM) and nipple-sparing mastectomy (NSM) [11,12,13] are often used although there have been concerns with oncologic safety and optimal surgical factors. The development of acellular dermal matrix (ADM) has made prepectoral and subpectoral/dual-plane implants feasible alternatives to submuscular implants for many patients [14], despite concerns of cost and complications such as infection. There is uncertainty about when ADM is required, and the potential adverse effects are not clear [15,16,17,18,19]. Autologous fat grafting, generally as a secondary procedure to improve contour irregularities and rippling, but also to supplement or replace the use of implants or autologous tissue is common, despite concerns about oncologic safety and sometimes suboptimal results potentially due to technical variations in the procedures. While potential benefits of breast reconstruction for women who desire this are generally acknowledged, uncertainty about techniques, timing, and the use of ADM and fat grafting prompted the development of this systematic review [20,21] and revised clinical practice guideline.

## 2. Materials and Methods

### 2.1. Background

The Program in Evidence-Based Care (PEBC) is an initiative of the Ontario provincial cancer system, Ontario Health (Cancer Care Ontario) supported by the Ontario Ministry of Health. The PEBC produces evidence-based and evidence-informed guidance documents using the methods of the Practice Guidelines Development Cycle [22,23]. This process includes a systematic review, interpretation of the evidence and draft recommendations by the Working Group, internal review by content and methodology experts, and external review by Ontario clinicians and other stakeholders. PEBC guideline recommendations are based on evidence of desirable and undesirable effects of an intervention and consider the certainty of the evidence. PEBC guideline development methods are described in more detail in the PEBC Handbook [24] and the PEBC Methods Guide [25].

The Surgical Oncology Program of Ontario Health (Cancer Care Ontario) identified a need to update the 2016 clinical practice guideline on breast reconstruction [26] and was a sponsor of this work. A Working Group with expertise in general surgery/surgical oncology, reconstructive/plastic surgery, radiation oncology, health research methodology, and patient representatives with personal or family experience with cancer was convened by the PEBC. The working group and sponsor determined the research questions and conducted a systematic review [20,21] to be used as the evidence base for the revised clinical practice guideline. Recommendations on the type of reconstruction (including the use of implants or various autologous flaps) were not updated and are available as an appendix in an extended version of this work [21].

### 2.2. Guideline Objective

This guideline was created to provide guidance on identifying which patients undergoing mastectomy due to breast cancer are candidates for reconstruction, the best timing of reconstruction (immediate or delayed), whether radiotherapy (RT) should influence timing, use of nipple-sparing mastectomy (NSM), the choice between prepectoral versus subpectoral implants, and use of autologous fat grafting and acellular dermal matrix (ADM) as part of the reconstruction process. For this document, “reconstruction” refers to immediate or delayed reconstruction of the breast mound and does not include the use of aesthetic flat closure.

### 2.3. Research Questions

1. What is the effect of patient factors (smoking status, body mass index, breast size, age), comorbidities (diabetes, hypertension), or oncologic factors (previous breast surgery, previous radiotherapy (RT) to the breast/chest, inflammatory breast cancer, skin involvement) on post-mastectomy breast reconstruction outcomes?
2a. In patients with breast cancer undergoing therapeutic mastectomy, is there a difference in outcomes in immediate versus delayed reconstruction for patients who do not receive RT?
2b. In patients with breast cancer undergoing therapeutic mastectomy, is there a difference in outcomes in immediate versus delayed reconstruction for patients who receive RT?
3a. In patients with breast cancer who are candidates for SSM/NSM and reconstruction, is there a difference in outcomes between NSM and SSM?
3b. In patients with breast cancer, do oncologic outcomes for NSM vary according to the criteria used in selecting patients for NSM (e.g., tumour to nipple distance) or how nipple/areolar involvement is assessed (e.g., clinical examination, mammography, MRI, other imaging, biopsy of areola/nipple/nipple core, frozen/intraoperative or permanent section)?
3c. In patients with breast cancer and NSM, what surgical factors have been reported that influence the rates of nipple viability or necrosis and retention of sensation after NSM?
4. Does the use of prepectoral implants for postmastectomy breast construction result in differences in outcomes than subpectoral implants?
5. After therapeutic mastectomy, do outcomes differ for breast reconstruction using human-derived ADM, synthetic absorbable matrix, or no scaffolding/matrix? Are there differences in outcomes between different human ADMs or different synthetic absorbable matrices?
6. What are the benefits and risks of autologous fat grafting (lipofilling) as an adjunct to breast reconstruction?

### 2.4. Target Population

Patients diagnosed with non-metastatic breast cancer who will undergo therapeutic mastectomy and are considering or have decided on reconstructive surgery. For purposes of this document, reconstruction includes both immediate and delayed reconstruction with implants and/or autologous tissue but does not include aesthetic flat closure.

### 2.5. Development Process

This guideline is based on the evidence in a systematic review completed in March 2025 [20,21]. The working group (authors) reviewed the evidence base, drafted the guideline recommendations, and addressed comments received during the document review process. The Working Group had expertise in general and plastic surgery, surgical oncology, radiation oncology, health research methodology, and lived experience with cancer as a patient or family member.

### 2.6. Literature Search

A literature search using OVID databases of Medline, Embase, EBM Reviews-Cochrane Central Controlled Trials, and EBM Reviews–Cochrane Database of Systematic Reviews was conducted in February 2023 and updated on 21 August 2024. The search contained terms for breast cancer, breast reconstruction, ADM, and fat grafting. There were 229 primary studies that met the inclusion criteria. A flow chart (PRISMA diagram) illustrating the search results and screening has been published with the systematic review [20].

### 2.7. Recommendation Development and Review

Based on the systematic review and supplemented by professional opinion, the Working Group developed clinical practice recommendations. The internal review consisted of reviews by an Expert Panel of 14 content experts and by the PEBC Report Approval Panel, a three-person panel with methodology expertise. All participants approved of the document; comments were considered by the Working Group in revising the document.

Two processes were used to obtain additional feedback on the approved draft guideline. Through the Targeted Peer Review, three individuals with content expertise were identified and asked to review and provide feedback. Through Professional Consultation, relevant care providers and other potential users of the guideline were contacted, and 31 provided feedback on the guideline recommendations through a brief online survey and additional comments. The Working Group considered all feedback in making final revisions.

## 3. Recommendations and Key Evidence

### 3.1. Patient Education and Preoperative Evaluation

#### 3.1.1. Recommendations

For women who have chosen or been recommended for therapeutic mastectomy, the following applies:

(a) The discussion of immediate or delayed breast reconstruction should be initiated at the time that mastectomy is offered by the general surgeon, breast surgeon, or surgical oncologist.
(b) For women seeking immediate breast reconstruction, preoperative evaluation should include a plastic surgeon.
(c) For women seeking immediate breast reconstruction who will potentially require adjuvant chemotherapy or radiotherapy (RT), a medical oncologist and/or radiation oncologist should be included in preoperative evaluation, either through a formal consultation or by a multidisciplinary cancer conference.
(d) Decisions around the contralateral breast should be jointly made by the patient and medical team, considering the patient’s family history and/or genetic profile if available and symmetry with the involved reconstructed breast, and include discussion of potential benefits and harms. The risk of new primary breast cancer may be a factor, and patients with risk factors for hereditary breast cancer should be referred for genetic assessment. The patient’s preferences and values must be considered, and an informed discussion is recommended prior to mastectomy, along with a recommendation by the surgeon conducting the mastectomy (e.g., general surgeon, breast surgeon, or surgical oncologist) or reconstructive surgeon (or consensus of these) for or against contralateral prophylactic mastectomy.

#### 3.1.2. Qualifying Statements

- Bilateral surgery is shown to increase complications risks.
- In the absence of genetic risk factors, contralateral mastectomy may decrease rates of new cancer but does not improve survival or recurrence.
- Contralateral mastectomy with reconstruction may give better aesthetic results in some patients. When contralateral mastectomy is not indicated, balancing procedures to the contralateral breast can also produce aesthetic and symmetrical results.

#### 3.1.3. Justification

- The recommendations are based on the expert opinion of the authors that women should be well informed about care options and that multidisciplinary preoperative evaluation is a quality indicator of multidisciplinary breast cancer care for women seeking reconstruction. Screening with magnetic resonance imaging (MRI) has been shown to detect synchronous contralateral breast cancer [27]. The American Society of Breast Surgeons consensus statement [28] states that contralateral mastectomy should be discouraged in average-risk women with unilateral breast cancer. A Canadian consensus statement recommends contralateral prophylactic mastectomy only in patients with *BRCA1/2* gene mutations or previous mantle field radiation, and consideration in case of other genetic mutations or where breast symmetry may be a major issue [29]. Ontario Health documents provide guidance for referral for genetic assessment and genetic testing [30,31].

### 3.2. Effect of Patient and Oncologic Factors

**Note 1**: While an individual factor may not be sufficient to rule out reconstruction, risks of complications for different factors may be additive and must be considered together (e.g., diabetes plus smoking plus obesity). A validated risk tool may be useful in evaluating overall risks. Further, some risk factors may alter the risk-benefit analysis for some types of reconstruction more than for other types. Factors that affect flap perfusion/circulation may preclude skin- or nipple-sparing mastectomy. The degree of comorbidity (e.g., grade of obesity, controlled vs. uncontrolled diabetes, current vs. former smoking and amount smoked) also needs to be considered.

**Note 2**: Published systematic reviews constitute the evidence base for Recommendations 1.2 through 1.6. The rationale for using higher level evidence was in part due to limited time and staffing resources, given the broad scope of this systematic review and is a usual trade-off. Furthermore, it is often the pragmatic choice to use higher-level evidence synthesis publications for these kinds of ‘factor’ questions, making use of work already performed by others to avoid duplication. As no systematic review was found that covered age adequately, Recommendation 1.1 on age is based on a new systematic review of this topic [20].

#### 3.2.1. Age

##### Recommendation

(a) Age on its own should not be used to determine whether to offer breast reconstruction to patients who are undergoing mastectomy as treatment for breast cancer. Competing risks of mortality and patient preferences should be part of the decision-making process; life expectancy and geriatric assessment may be considered.

##### Qualifying Statements

- Comorbidities including heart diseases and diabetes tend to increase with age and may affect suitability for operation or wound healing.
- While some older patients may place less importance on breast reconstruction, it should not be assumed for all, as individual preferences will vary.
- Some patients of any age may not want reconstruction and prefer mastectomy alone, and it should not be assumed that all younger patients will want reconstruction.

##### Key Evidence

- A single institution study by Kim et al. [32] considered age as a continuous variable. Odds ratios (OR) per year of age increase for mastectomy skin flap/nipple necrosis was OR = 1.02 (95% CI = 1.01 to 1.03, *p* < 0.001), infection OR = 1.01 (95% CI = 1.00 to 1.03, *p* = 0.038), seroma OR = 1.02 (95% CI = 1.00 to 1.03, *p* = 0.024), and hematoma OR = 1.01 (95% CI = 1.00 to 1.03, *p* = 0.14). Using the BREAST-Q, Satisfaction with Breasts was lower in older patients, while Psychosocial Well-Being was higher, and there was no difference in Physical Well-Being-Chest, nor Sexual Well-Being.
- A study of pedicled flap reconstruction using the American College of Surgeons National Surgery Quality Improvement Program (ACS-NSQIP) database [33] also used age as a continuous variable. For complications within 30 days of surgery, they reported odds ratios (OR) for overall complications of 1.010 (95% CI = 1.004 to 1.006, *p* = 0.002), severe complications OR = 1.043 (95% CI = 1.019 to 1.068, *p* < 0.001), and wound complications OR = 1.009 (95% CI = 1.000 to 1.018, *p* = 0.053). They concluded the effect of age does not have strong predictive power and may not be clinically relevant on its own.
- The Mastectomy Reconstruction Outcomes Consortium Study (MROC) compared outcomes according to three age groups. For patients aged > 60 versus <45 years old, they reported OR = 1.46 (95% CI = 0.99 to 2.15, *p* = 0.059) for any complications and OR = 1.43 (95% CI = 0.93 to 2.18, *p* = 0.101) for major complications. For patients aged 45–60 versus <45 years old, they reported OR = 1.23 (95% CI = 0.93 to 1.62, *p* = 0.140) for any complications and OR = 1.16 (95% CI = 0.85 to 1.57, *p* = 0.349) for major complications. There were higher BREAST-Q scores for Sexual Well-Being for both implants and autologous reconstruction in older women than for those < 45 years old, and for Psychosocial Well-Being and Physical Well-Being with autologous reconstruction.

#### 3.2.2. Body Mass Index, Smoking, Diabetes, Hypertension

##### Recommendation

(a) High body mass index (BMI), current and prior smoking status, diabetes, and hypertension are risk factors for complications and poorer outcomes but should not be used as absolute contraindications to reconstruction. It is recommended that uncontrolled diabetes be treated and that patients cease smoking at least several weeks prior to surgery and until the incisions have healed.
(b) Reconstruction should be presented as an option, and patients informed that risks of specific complications such as skin or nipple necrosis and reconstructive failure are higher than in patients without risk factors.

##### Qualifying Statements

- Obesity is often defined as BMI > 30 kg/m^2^, although particularly in Asian countries a lower cut-off (25 kg/m^2^) is often used. Risks of complications increase with BMI above these thresholds on a continuum. Incisions and reconstruction techniques may need to be altered and include contralateral reduction if matching of the breasts is considered important to the patient. The amount of tissue removed, and repositioning of the nipple may result in ischemic complications and decreased sensation.
- In patients with multiple risk factors or comorbidities, the potential effect of all combined must be considered. A validated risk assessment tool may be used.

##### Key Evidence

- Diabetes. Two moderate-quality systematic reviews on diabetes found increased complications: one [34] found higher rates of overall complications, surgical complications, implant loss/failure, infection, and skin necrosis, while the other [35] found a higher rate of wound dehiscence.
- Smoking. A systematic review [36] found that ever smoking was a risk factor for postoperative complications, donor site complications, infection, and fat necrosis. The review was rated as low quality due to non-reporting of risk of bias of individual studies but strengthened due to study size (26 studies and >20,000 patients) and consistency of results between studies.
- BMI or Obesity. A moderate-quality systematic review [37] looked at the effect of obesity (BMI > 30 kg/m^2^) in 30 studies and found higher rates of surgical complications (relative risk [RR] = 2.29, 95% confidence interval [CI] = 2.19 to 2.39, *p* < 0.00001), as well as higher rates of fat necrosis, seroma, partial or total flap failure, wound dehiscence, wound infection, hernia, medical complications, and return to operating room. In a subset of four studies that reported surgical complications by obesity class, surgical complications increased with class of obesity: class I (30 to 34.9 kg/m^2^) RR = 1.32; class II (35 to 39.9 kg/m^2^) RR = 1.84; and class III (>40 kg/m^2^) RR = 1.66. Another moderate-quality systematic review [38] compared autologous versus implant reconstruction in patients with obesity and found that autologous reconstruction resulted in lower infection, hematoma, seroma, and reconstructive failure; no difference in skin necrosis or wound dehiscence; and increased rates of deep vein thrombosis and pulmonary embolism compared to implant-based reconstruction.
- Hypertension. A systematic review [39] on complications after breast reconstruction found that hypertension increased the risk of any complication, major/re-operative complications, 90-day readmissions, and infection. The studies included for other questions in our systematic review suggest the effect of hypertension to be low and therefore often not statistically significant in the small studies. Most studies did not define this variable and may have used different cut-offs. The review [39] is rated low due to non-reporting of risk of bias of individual studies but strengthened due to a large number of studies and patients (33 studies with over 100,000 patients, of which 7 studies reported any/total complications by hypertension status).

#### 3.2.3. Breast Size

##### Recommendation

(a) Pre-mastectomy breast size and desired reconstructed breast size may influence the type of reconstruction, complication rates, and cosmetic/aesthetic results; however, these factors should not determine whether to perform reconstruction. Patients and surgeons should discuss the risks and benefits of various procedures.

##### Key Evidence

- Several of the included studies about NSM under Question 3 in the Systematic Review [20] noted that greater breast size or mastectomy weight, initial expander fill volume in two-stage implants, and final implant size in direct-to-implant reconstruction were risk factors for necrosis. These factors may affect perfusion, with the latter two putting more tension on the skin. Modifications to reconstructive procedures using lower initial fill volumes and minimizing the use of direct-to-implant reconstruction have been used by some groups.

#### 3.2.4. Previous Surgery

##### Recommendation

(a) Abdominal scars, previous abdominal surgery, and previous breast augmentation are not contraindications to breast reconstruction but may influence the surgical and reconstructive planning and type of reconstruction performed.

##### Key Evidence

- A systematic review of moderate quality on the effect of prior abdominal surgery on abdominally based flap reconstruction [40] found a small increase in donor-site delayed wound healing.
- A systematic review of the effect of pre-existing abdominal scars on reconstruction using abdominal flaps [41] suggests there may be a small increase in flap complications or flap loss; however, differences were not statistically significant. There were higher donor-site complications than in control patients without abdominal scars. The authors suggest constraints of the scar may be overcome by technical modifications and the use of computed tomography angiography to assess the presence of perforator vessels if there is uncertainty. This review was rated low quality due to the absence of risk of bias assessment and study protocol.
- Another low-quality systematic review [42] based on six studies found similar rates of complications in patients with and without prior breast augmentation (early complications odds ratio [OR] = 1.57, 95% CI = 0.94 to 2.64; overall complications OR = 1.23, 95% CI = 0.76 to 2.00).

#### 3.2.5. Neoadjuvant Chemotherapy

##### Recommendation

(a) Patients who received neoadjuvant chemotherapy (NACT) should be assessed and considered for reconstruction in the same manner as patients without NACT.

##### Key Evidence

- A high-quality systematic review [43] compared reconstruction in patients with and without NACT, and did not find a statistically significant difference in overall complications (RR = 0.91, 95% CI = 0.74 to 1.11, *p* = 0.34), flap loss (RR = 0.94, 95% CI = 0.46 to 1.94, *p* = 0.87), hematoma (RR = 0.99, *p* = 0.97), or wound complications (RR = 1.15, *p* = 0.22). There was an increase in implant/expander loss (17.4% vs. 11.3%, RR = 1.54, 95% CI = 1.04 to 2.29, *p* = 0.03). Most studies did not report adjusted risk estimates, and patients with NACT often had moreadvanced disease.

#### 3.2.6. Radiotherapy

##### Recommendation

(a) Adjuvant radiotherapy (RT) should not be considered as a contraindication to either implant-based or autologous reconstruction. Patients should be informed that adjuvant RT is associated with increased reconstructive complications and that these are greater in expander/implant reconstruction than with autologous reconstruction.

##### Qualifying Statements

- The type of complications varies between implant and autologous reconstruction. Autologous reconstruction may be preferred in patients at higher risk of implant-related complications.
- Timing of RT with respect to reconstruction may influence the degree or profile of complications and is not addressed here. The timing of reconstruction and use of RT is partially addressed in Recommendation 2.

##### Key Evidence

- Four systematic reviews on the effect of post-mastectomy radiotherapy (PMRT) on implant-based breast reconstruction [6,44,45,46] found increased rates of complications with PMRT. Adjuvant RT also resulted in lower patient satisfaction and aesthetic results. Two reviews were judged to be of moderate quality and two of low quality.
- A high-quality systematic review of PMRT in conjunction with autologous flap reconstruction [9] found higher rates of fat necrosis, secondary surgery, and volume loss. Observer-reported cosmetic results were lower with PMRT. It was noted that there are higher risks with PMRT, but these are not necessarily clinically significant.

#### 3.2.7. Justification for Recommendations 3.2.1 to 3.2.6

- The authors consider that the potential psychological and quality of life (QoL) benefits of reconstruction may outweigh the risks of complications related to factors in Recommendations 1.1 to 1.6 for some patients. While rates of complications are moderately increased, they are generally manageable and often modifiable. Risks of complications and reconstructive outcomes vary depending on patient and disease characteristics and type of reconstruction, and decisions should be individualized during consultation between the patient and the medical team. Considering implant-based reconstruction in the immediate setting could be particularly beneficial for patients with limited options for autologous reconstruction due to slim body habitus. Patients valued being part of the decision-making process. Consideration of reconstruction, including a discussion of risks and benefits, may ensure equal access for patients with manageable comorbidities or conditions.
- The recommendation on age is consistent with The European Society of Breast Cancer Specialists/International Society of Geriatric Oncology guideline [47] for older patients (age ≥ 70 years) and the published evidence that indicates no significant differences in most outcomes by age. Breast reconstruction may improve QoL for patients of any age. Older patients have a low rate of reconstruction compared with younger patients. While this is partially related to comorbidities or patient choice, it is also due to lack of access including not being presented with the option [48]. Considering reconstruction for patients of all ages would increase equity.

### 3.3. Immediate Versus Delayed Reconstruction

#### 3.3.1. Recommendation

(a) For patients desiring breast reconstruction, both immediate and delayed reconstruction may be considered.
(b) When delayed reconstruction occurs after radiotherapy (RT), reconstruction should occur at least 6 months after completion of RT, or longer if the irradiated site is still acutely tight, inflamed, and prone to complications.

#### 3.3.2. Qualifying Statement

- The preferred timing of reconstruction will depend on factors such as patient preferences, type of mastectomy, skin perfusion, comorbidities, pre-mastectomy breast size, and desired reconstructive breast size.
- Immediate reconstruction may provide greater psychological or QoL benefits for some patients.
- Access to and resources for delayed breast reconstruction can be very lengthy in parts of Ontario and immediate reconstruction would avoid being on a lengthy waitlist.

#### 3.3.3. Key Evidence

##### Without RT

- The Mastectomy Reconstruction Outcomes Consortium (MROC) study [49] found that patients with delayed reconstruction scored significantly lower on most QoL scales prior to reconstruction. Two years postoperatively there were no significant differences in adjusted patient-reported outcomes (PROs) between immediate and delayed groups.
- Shammas et al. [50] used claims codes to study immediate, delayed, and staged (delayed-immediate with spacer/expander at the time of mastectomy) free-flap reconstruction and associated complications within 90 days of either operation. After adjustment, the relative risk of at least one complication was lower for immediate versus delayed (RR = 0.78, 95% CI = 0.68 to 0.88, *p* < 0.001), immediate versus staged (RR = 0.60, 95% CI = 0.53 to 0.67, *p* < 0.001), and delayed versus staged (RR = 0.77, 95% CI = 0.67 to 0.88, *p* < 0.001). Part of the increased complications in delayed and staged groups appears to be due to additive complications as a result of two operations (mastectomy and later reconstruction) compared with one operation in the immediate setting.
- Huang et al. [51] found similar complications in immediate and delayed groups with deep inferior epigastric perforator (DIEP) flap reconstruction, except for higher breast skin necrosis in the immediate group. Authors suggest lower rates in the delayed group may be due to additional time to revascularize and heal after mastectomy. Prantl et al. [8] studied DIEP flap reconstruction and found similar complication rates, including flap loss, between immediate and delayed reconstruction.

##### With RT—Implants

- Ulrikh et al. [52] found similar rates of complications for immediate two-stage implants with PMRT to the expander and for PMRT then delayed implants.

##### With RT—Autologous

- A report of the MROC study compared mastectomy and immediate autologous reconstruction followed by PMRT versus mastectomy then PMRT then delayed reconstruction [53]. The difference between the immediate and delayed groups for any breast complication was not significant (OR = 0.64, *p* = 0.442 at 1 year; OR = 1.14, *p* = 0.848 at 2 years). Prior to reconstruction (before mastectomy in the immediate group but after mastectomy for the delayed group), the immediate group had better scores on the BREAST-Q questionnaire for Satisfaction with Breasts (59.5 vs. 36.3, *p* < 0.001), Psychosocial Well-Being (66.1 vs. 50.0, *p* < 0.001), and Sexual Well-Being (52.1 vs. 29.8, *p* < 0.001) and differences are likely due to presence/absence of breasts. After reconstruction, there were no significant differences in PROs at 1 year.
- Hassan et al. [54] compared staged (skin-sparing; immediate expander/spacer then reconstruction) to delayed microvascular (autologous) reconstruction. PMRT, when used, was to the expander in the staged group and prior to reconstruction in the delayed group. Complications were generally similar except for seroma (higher in the delayed group without PMRT) and infection (higher in the delayed group with PMRT). Expander loss, which by definition can only occur in the staged group, was 10.6%; the implications of this on treatment were not reported. The staged group had fewer flap-related complications but higher overall complications when expander loss was factored in (34.2% vs. 25.5%). The overall time from mastectomy to reconstruction was longer in the delayed group.
- A review by Koesters and Chang [55] reports on the timing of flap reconstruction (immediate or delayed) in patients with PMRT. They noted heterogeneity in outcomes between studies and that RT regimes vary over time and between institutions. Using modern RT, outcomes may be more similar and complications lower than suggested by previous studies and reviews. Immediate reconstruction has benefits in avoiding the psychosocial effects of an absent breast and may be preferred, although delayed reconstruction is also reasonable.

##### Delay Between PMRT and Reconstruction

- Studies included for other questions offer guidance as to current practice. They were consistent in waiting at least 3 months to avoid the period of acute radiation injury for both delayed autologous reconstruction [7,51,56] and for expander-implant exchange after PMRT to the expander [57,58,59,60]. While 3 months was considered an absolute minimum, and 3 to 6 months was sometimes used [7,57], a minimum of 6 months was more common [51,59,60,61].
- While a systematic review was not conducted on this sub-question, three comparative studies are informative. One study [56] included for other topics reported vascular complication rates (intraoperative arterial and venous thrombosis, intraoperative technical difficulties during anastomosis, delayed vascular complications) when autologous reconstruction was conducted at various intervals after PMRT. Vascular complications were 66% with an interval < 3 months after completion of PMRT, 20% with a 3-to-12-month interval, 14% with a 12-to-120-month interval, and 19% with >120 months. The probability of a difference between the <3 months and 12 to 120 months groups was *p* = 0.06. Two other studies by the same clinical group [62,63] reported reconstructive failure according to the interval between PMRT to the expander and expander-implant exchange. These prompted an increase in this interval from 3 months to 6 months as their general protocol, including in two of the studies [59,60] reported for other questions in this review.
- A review by Koesters and Chang [55] indicates that many surgeons use clinical assessment of skin quality and laxity, avoiding a site that is still acutely tight, inflamed, and predisposed to complications, instead of a fixed delay between RT and reconstruction.

#### 3.3.4. Justification

- The recommendation is based on better short- to medium-term QoL and psychological outcomes with immediate construction, unsuitability of immediate reconstruction for some patients, and a small increase in risk of total complications with number of operations. The benefits of reconstruction are considered to be well-established and therefore were outside the scope of the systematic review. It is the consensus of the authors, and supported by the MROC study, that worse psychological status and QoL may occur in the period between mastectomy and reconstruction, and this is reduced in immediate reconstruction. The benefits of immediate reconstruction need to be balanced against the fact there are sometimes clinical reasons such as poor perfusion, contraindications to longer operation combining mastectomy and reconstruction in immediate reconstruction, or patient uncertainty regarding reconstruction that may make delayed reconstruction preferable. In the systematic review [20], differences in complications between immediate and delayed reconstruction were small and inconsistent between studies, suggesting that patient and surgical factors may have a greater effect on complications than timing. Data on comparing complications have a high risk of bias and we only conclude that risks are similar and usually manageable. The decision of timing should be a joint decision between patients and physicians, taking into account individual situations, values, and risk profiles.
- Minimizing delay for reconstruction is important for patients. While optimal timing of reconstruction after PMRT to avoid complications has not been established, the authors support 6 months as was frequently used in the included studies. This may be modified by clinical assessment of skin quality. Fat grafting (see Recommendation 6) may be of benefit.

### 3.4. Nipple-Sparing Mastectomy

#### 3.4.1. Nipple-Sparing Versus Skin-Sparing Mastectomy

##### Recommendations

(a) In patients who are candidates for skin-sparing mastectomy (SSM) and without clinical, radiological, and pathological indications of nipple-areolar complex (NAC) involvement, nipple-sparing mastectomy (NSM) is recommended provided it is technically feasible and acceptable aesthetic results can be achieved.
(b) Patients should be informed that in the case of tumour involvement of subareolar tissues/margins based on pathologic analysis, or of NAC necrosis not responding to treatment, the nipple or NAC may need to be excised.
(c) The patient should be involved in the decision between NSM and SSM. The patient should be informed, along with reasons, if NSM is considered inappropriate and not being offered.

##### Qualifying Statements

- Comorbidities, larger breast size, and ptosis are risk factors for poor perfusion and subsequent skin flap and/or NAC necrosis. Reduction in breast size and repositioning of the nipple may require different incision locations. Blood supply to the skin flap/NAC may be improved with delayed reconstruction, staged mastectomy, or surgical delay when the oncologic treatment timeline allows.
- Discussion of tattooing or nipple reconstruction with realistic restorative areola tattooing needs to be a discussion for psychological and physical well-being in patients for which NSM is not suitable.

##### Key Evidence

- Five studies [64,65,66,67,68,69] suggest that both NSM and SSM have similar oncologic results when pathologic analysis of retroareolar/excised tissue does not show tumour involvement. Cho et al. [67] used both propensity-score matching and Kaplan–Meier and Cox proportional hazard regression and found 5-year disease-free survival (DFS) of 89.1% versus 90.7% (hazard ratio [HR] = 1.15, 95% CI = 0.65 to 2.03, *p* = 0.64), 5-year overall survival (OS) of 97.3% versus 97.4% (HR = 1.05, 95% CI = 0.34 to 3.20, *p* = 0.93), and local recurrence-free survival (LRFS) of 95.8% versus 96.8% (HR = 1.22, *p* = 0.68). Kim et al. [68] adjusted for patient and tumour characteristics and found similar DFS (HR = 1.004, 95% CI = 0.52 to 1.93, *p* = 0.991) and OS (HR = 0.866, 95% CI = 0.26 to 2.85, *p* = 0.813).
- Racz et al. [70] found better BREAST-Q questionnaire scores on Sexual Well-Being for the NSM group (mean 64.5 vs. 58.0, *p* = 0.002, multivariable *p* = 0.033).
- Delayed reconstruction, staged mastectomy, or surgical delay have been used to improve blood supply to both the skin flap and the NAC [71,72,73,74,75,76].

##### Justification

- The authors judge that there is moderate certainty of evidence that survival is similar after NSM compared with SSM. It is well-established that NSM has positive psychological and QoL benefits (evidence not reviewed) and is preferred by patients. We therefore recommend NSM instead of SSM in cases without NAC involvement provided this is surgically feasible and suitable aesthetic results can be obtained.

#### 3.4.2. Patient Selection and Assessment of Nipple-Areolar Complex (NAC) Involvement

##### Recommendations

(a) Nipple-sparing mastectomy (NSM) can be considered in all patients with non-metastatic, non-inflammatory breast cancer without clinical signs of nipple involvement (bloody or pathologic nipple discharge, nipple retraction, Paget disease) and no nipple involvement by imaging and where it is surgically feasible and suitable aesthetic results can be obtained.
(b) An oriented subareolar sample must be obtained for pathologic evaluation. A sample of ducts from the nipple or complete nipple coring (total skin-sparing mastectomy [TSSM]) may be considered.
(c) In cases where specimens taken from the area immediately under or within the nipple are found involved by tumour, but the areola is not involved, nipple excision alone (i.e., areola-sparing mastectomy [ASM]) may be conducted provided clear margins are obtainable.
(d) Involvement of areolar skin not extending to the nipple may be treated as for other skin cancers and excised with clear margins.
(e) We recommend against intraoperative/frozen section pathologic analysis. Treatment decisions should be based on definitive/final pathology results.
(f) The patient should be informed that NAC or nipple excision is the standard treatment when the subareolar area is found to be involved with tumour on final pathologic analysis; the final decision should be made by the patient and surgical team. Planned RT may be a factor in the decision.
(g) Prior to a planned NSM, patients should be informed and consent to NAC or nipple removal if intraoperative surgical findings are indicative of cancer that cannot be resected without NAC or nipple excision.

##### Qualifying Statements

- SSM, ASM, and NSM aim to balance eliminating negative oncologic outcomes with maintaining a viable skin envelope. Differences in operative procedures and criteria may contribute to variations in outcomes between studies.
- While intraoperative frozen section analysis was used in several of the published studies, it is less accurate and may result in false positives (and unnecessary NAC excision) or false negatives (with involved NAC retained). When subareolar tissue is found involved by tumour on intraoperative/frozen section analysis, re-excision to obtain clear margins may be conducted as an alternative to immediate NAC excision.
- Studies did not use a uniform definition of pathologies that would require NAC excision. Atypia and lobular carcinoma in situ (LCIS) in subareolar samples were not criteria to conduct NAC excision in several studies; frozen section analysis is not a reliable method of assessing epithelial cell atypia and is often misidentified in frozen section analysis.

##### Key Evidence

• Studies in the systematic review [20] found that smaller tumour-to-nipple distance (TND; <5 cm, <2 cm or <1 cm) measured by MRI is a risk factor for NAC involvement but did not predict worse oncologic outcomes [11,77,78,79,80,81]. TND may influence surgical strategies but should not be used to rule out NSM or ASM.
• Recurrence, survival, and necrosis rates for most studies in the review [20] were similar to those expected for other types of breast cancer treatment. Worse outcomes are often due to the selection of patients with more advanced cancers or different operative techniques (see Question 3c).
• Nipple excision or ASM was used in several of the included studies [12,82,83,84,85,86,87]. Shanno et al. [85] found no significant difference in periareolar recurrence for patients with nipple excision compared with those with NAC excision (2/64 or 3.1% vs. 0/34). Simmons et al. [88] found that of 217 mastectomy patients, 23 had nipple involvement and 2 had areola involvement (both stage III central tumours), suggesting the areola can often be preserved even with nipple involvement. They indicate that the areola does not contain breast parenchymal ducts. Pacella et al. [89] indicate that in NSM it is the ductal tissue inside and immediately below the nipple that is the oncologic concern, while the areola consists of pigmented skin with skin adnexal glands that do not connect by ducts to breast tissue, and therefore ASM may be used and produces better aesthetic outcome than SSM.
• Several studies indicate that frozen section/intraoperative pathology is less accurate and is not used by their group [80,84,85,86,87,90,91,92,93,94]. Spoor et al. [95] found sensitivity and specificity of frozen section analysis were 75.2% and 98.5%, respectively, compared with definitive histopathology, while Zhu et al. [12] reported a sensitivity of 81.8% and specificity of 95.3%.
• The systematic review [20] found that local recurrence (LR) in the NAC in 41 studies varied from 0% to 6%, with a median of 0.5% and an average of 1.2%.
○ Of 17 studies with no NAC recurrence, 8 were from USA, 6 from Italy, and 1 each from Brazil, Germany, and Canada. The nine highest values (≥1.9%) were from Korea, Japan, and China. It is not known whether these geographic differences are due to differences in surgical technique or patient populations.
○ In studies with pathologic analysis of only retroareolar samples, the median and average LR in the NAC were 1.4% and 1.6%. In studies that sampled ducts or cored the nipple (sometimes referred to as TSSM), the median and average LR in the NAC were 0 and 0.5%. There were three studies with recurrence conducted in East Asia and 13 studies without NAC recurrence.
• The median rate of total nipple necrosis in 44 studies was 1.9% (mean 2.8%). The review found that TSSM did not increase rates of total nipple necrosis [20].

##### Justification

- Sparing the NAC (or part of the NAC) whenever oncologically safe is important to patients. Recommendations for joint decision-making are the consensus of the authors based on the need for equity and mutual respect. The literature review indicates that the absence of clinical/radiological nipple involvement is an appropriate selection criterion, provided that excision of the NAC, nipple, or (partial) areola takes place if clear margins cannot be obtained. There is a lack of consensus as to the extent of excision during mastectomy and number/location of samples for pathologic assessment before resorting to NAC/nipple/areolar excision. The authors consider retroareolar sampling to be appropriate to balance oncologic safety and nipple preservation. Sampling or removal of ducts within the nipple (nipple coring or TSSM) is also reasonable as this may slightly reduce NAC recurrence, but with increased risk of sensation loss and of necrosis due to reduced perfusion of the nipple. Our review did not indicate an increased risk of necrosis with TSSM, although the TSSM study authors implemented procedures to reduce necrosis such as avoiding single-stage reconstruction [90,96]; they did acknowledge a potential for decreased sensation and that this may be more technically challenging surgery. Surgical factors (see following recommendations) are also important. Some consider removing ducts in the nipple unnecessary as terminal duct lobular units are more likely at the nipple base and rare in the tip or papilla [97,98] and therefore increases the risk of adverse events without additional benefit.

#### 3.4.3. Surgical Factors in Nipple-Sparing Mastectomy: Evidence Note

Authors of studies included in the systematic review indicated that incision location, nerve preservation, skin tension, thermal damage in dissection, and operative planning are important surgical factors for outcomes of nipple viability/necrosis and sensation after NSM. Several studies reported comparative data on incision location and a few on sensation. The study authors presented the other factors as generally accepted or based on their accumulated experience. These were not the topics of comparison/investigation and are presented in the following as important to be considered but without specific recommendations or optimal approaches.

#### 3.4.4. Surgical Factors in NSM: Incision Location

##### Recommendations

(a) Periareolar incisions (including hemi-periareolar) should be avoided unless there are oncologic or other specific reasons for their use. Periareolar incisions, if used, should encompass no more than one-third of the areolar circumference.
(b) To reduce nipple necrosis, inferolateral (lateral inframammary fold) or inframammary fold (IMF; central inframammary fold) incisions are preferred.
(c) In the case of previous breast surgery with scars, it may be preferable to reoperate using the same incision. This should be determined on a case-by-case basis.

##### Qualifying Statements

- Re-excision using the same incision location as for previous surgery is sometimes used to avoid multiple sets of scars that may have a negative aesthetic impact. As the previous incision already disrupted blood supply in that area, reusing the same scars may also cause fewer additional complications.
- Inferolateral and IMF have the least necrosis associated with them. Inferior radial (vertical inferior) or lateral radial (horizontal radial) incisions have been reported as resulting in intermediate levels of nipple necrosis.
- As indicated in Question 5 on acellular dermal matrix (ADM) use, inferolateral incisions may be preferred for subpectoral implants without ADM. Some have suggested that incisions in the IMF may not allow for enough support for implants unless ADM is used. There is not enough evidence in this regard to make a recommendation, but the type and location of the implant may influence the incision location.

##### Key Evidence

- Moo et al. [99] reported necrosis by incision type, and calculated adjusted ORs (aOR) relative to lateral radial as reference: 12.1% lateral IMF (OR = 0.41; aOR = 0.35, 95% CI = 0.17 to 0.7, *p* = 0.003); 19.0% central IMF (OR = 0.64; aOR = 0.54, 95% CI = 0.19 to 1.39, *p* = 0.200); 29.8% lateral radial (OR = 1.0 as reference); 37.3% inferior periareolar/lateral extension (OR = 1.25; aOR = 1.24, 95% CI = 0.52 to 2.93, *p* = 0.600); 41.2% superior periareolar/lateral extension (OR = 1.38; aOR = 1.59, 95% CI = 0.65 to 3.92, *p* = 0.300).
- Lin et al. [100] reported relative rates of nipple necrosis by incision type compared to inferolateral inframammary fold (reference, 1.0): vertical inferior OR = 2.124 (95% CI = 0.453 to 9.944, *p* = 0.339); extension of prior incision OR = 2.98 (95% CI = 0.657 to 13.511, *p* = 0.157); horizontal radial OR = 3.823 (95% CI = 1.081 to 13.515, *p* = 0.037); and periareolar OR = 14.235 (95% CI = 6.248 to 32.435, *p* < 0.001).
- Park et al. [101] reported complete nipple necrosis of 3.4% with IMF incision, 11.3% with radial incision, and 21.3% with periareolar incision (multivariate vs. IMF; OR = 3.628, 95% CI = 1.596 to 8.250, *p* = 0.002).
- Lai et al. [102] found that periareolar incisions had a higher risk of NAC ischemia or necrosis compared with upper outer radial incisions (OR = 5.33, 95% CI = 1.81 to 15.67, *p* < 0.01).
- The group at Massachusetts General Hospital (Boston) prefers use of an inferolateral IMF incision [100,103,104] as there are less complications including nipple and skin necrosis.
- Surgeons at the University of California use primarily IMF incision or superior periareolar incision encompassing less than one-third of the areolar circumference [90,93,105,106], with others being much less frequently used. For patients who are candidates for either type of incision, the inframammary location is preferred due to a much lower rate of NAC complications (7.4% vs. 25%, OR = 4.2, 95% CI = 1.5 to 11.3, *p* = 0.003) [93]. Wong et al. at the University of Kentucky also use inferolateral incisions for TSSM [107].
- A group of plastic surgeons from various institutions in the USA published a resource in 2024 to guide breast and plastic surgeons in mastectomy techniques that preserve nerves, including applied anatomy of breast innervation and steps to incorporate nerve-sparing mastectomy and breast neurotization [108]. For most patients, they prefer an inferolateral IMF incision, although a Wise patten reduction incision may be beneficial in patients with grade 3 ptosis or macromastia. The inferolateral incision allows better visualization and exposure of the lateral intercostal nerves and good cosmetic results, while an inferior vertical incision may be useful, especially for surgeons with less experience in neurotization. Further details are in the guide.

##### Justification

- Evidence is strong that the location of incisions influences the risk of nipple necrosis.

#### 3.4.5. Surgical Factors in NSM: Nerve Preservation

##### Recommendation

(a) Selection of incision sites should take into account both preservation of blood supply and minimizing nerve damage.

##### Qualifying Statements

- Priority should be to minimize nerve damage and optimize conditions for nerve regeneration. Partial sensation, while much lower than prior to mastectomy, may be maintained in some patients. Reconnecting nerves is sometimes attempted in autologous flap reconstruction.

##### Key Evidence

- Studies cited earlier in Recommendation 3.3 regarding incision site selection mostly focused on minimizing necrosis but may be applicable to sensation as well.
- Black et al. [109] studied 192 patients with NSM. They found better sensory recovery in 106 patients with autologous reconstruction with neurotized DIEP flaps than in 86 patients with immediate expander-implant reconstruction. Sensory recovery was experienced in both groups, although the autologous cohort had greater improvement in five of nine regions at 1 year and seven of nine regions at 4 years postoperatively.
- Other studies meeting our inclusion criteria were limited; they noted partial sensation after reconstruction. Collaboration of breast and plastic surgeons prior to surgery is beneficial [110,111].
- A group of plastic surgeons from various institutions in the USA published a resource in 2024 to guide breast and plastic surgeons in mastectomy techniques that preserve nerves, including applied anatomy of breast innervation and steps to incorporate nerve-sparing mastectomy and breast neurotization (including when allografts are not available) [108]. For most patients, they prefer an inferolateral IMF incision, although a Wise patten reduction incision may be beneficial in patients with grade 3 ptosis or macromastia. The inferolateral incision allows better visualization and exposure of the lateral intercostal nerves and good cosmetic results, while an inferior vertical incision may be useful, especially for surgeons with less experience in neurotization. Further details are in the guide.

##### Justification

- Historically, the reconstruction goal was to only restore the appearance of the breast; however, lack of sensation has resulted in injuries such as severe burns, and psychological/QoL issues such as not being able to feel a hug, not being able to feel their own or a partner’s touch, feeling like the breast is not part of them, and lower sexual well-being. While oncologic safety is always the first priority, recent studies suggest adaptations in the mastectomy technique to retain or allow regeneration of partial sensation in some patients is possible and may improve QoL. From a patient perspective, this is an important consideration. Planning incisions including identifying nerves to minimize damage is the first step. Connecting nerves in autologous flaps to those in the mastectomy site may be possible in some patients but is not yet standard of care. While results appear promising with nerve allografts, studies are limited, expertise may not be available in Ontario, and nerve allografts are not currently funded. The authors judge the evidence insufficient to make a recommendation regarding nerve allografts. Patients must be advised that even with most advanced techniques there are no guarantees of success, sensation will be different and less than before mastectomy, and it will require several months and up to 2 years before maximum recovery.

#### 3.4.6. Surgical Factors in NSM: Skin Tension

##### Recommendation

(a) When nipple-sparing mastectomy (NSM) is followed by immediate expanders or implants, excess tension should be avoided as it may interfere with blood flow and lead to necrosis.

##### Key Evidence

- While there was no direct comparison, several authors commented on the effect of skin tension on necrosis. The viability of the NAC depends on the preservation of the blood supply to the nipple, ducts, and surrounding skin [112]. Immediate fixed-volume implants may put more tension on the skin flaps than tissue expander that are gradually inflated. Ahn et al. [113] indicated that higher skin tension may interfere with blood flow and be the cause of higher rates of necrosis with direct-to-implant and autologous reconstruction. When using tissue expanders, they prefilled them to approximately one-third of the final volume to minimize tension. Holland et al. [93] also ensured tissue expanders were filled to prevent significant wrinkling but not to an extent that would place tension on the closure or pressure on the overlying skin. Park et al. [101] noted that breast volume or weight is a risk factor for necrosis and that this may be due to increased skin tension and longer distance from the blood source to the nipple, which both decrease nipple blood supply. Pek et al. [114] used a skin paddle incorporated into the closure if excessive tension was anticipated. Ng et al. [115] note that Asian women have relatively smaller breast envelopes and therefore increased potential for skin tension leading to nipple necrosis than in Western counterparts. Warren Peled et al. also advocate using only minimal expansion of thin mastectomy skin flaps, with two-stage expander implants being preferred to minimize ischemic complications and necrosis [90].

##### Justification

- Studies comparing different degrees of skin tension in NSM were not found in the systematic review. It is the consensus of the authors, and consistent with procedures and comments in several of the included studies, that skin tension should be minimized by limiting the size of implants or initial inflation of expanders to maintain NAC and skin viability and prevent necrosis.

#### 3.4.7. Surgical Factors in NSM: Thermal Damage in Dissection

##### Recommendation

(a) Care should be taken to minimize thermal damage to the skin, blood vessels and nerves.

##### Key Evidence

- Many studies in our systematic review did not specify dissection methods and none made a direct comparison. The issue of avoiding thermal damage was mentioned in six of the included publications, as well as other reviews. Outcomes may depend on the surgical skills and techniques.
- Sharp dissection has been recommended to avoid thermal injury from electrocautery that may damage nerves [110,111] and damage the skin envelope [81] and vessels in the 1 to 3 mm layer of dermal tissue and result in necrosis [116]. Three studies in the review indicated electrocautery was used only for bleeding control, or at low settings to minimize thermal injury [20].
- Ng et al. [117] found that a switch from electrocautery to sharp dissection with tumescence resulted in a decrease in full-thickness necrosis from 12.8% to 1.3% and partial-thickness necrosis from 33.3% to 13.0% and reduction in operating time from 202.9 to 183.5 min. This was limited by the small number of patients (66 patients and 116 breasts).
- Tumescence is sometimes used together with sharp dissection to establish a bloodless plane for dissection [118]. Contradictory results have been found in various studies. Tumescence is reported to interfere with indocyanine green (ICG) angiography [119] and some other assessments of perfusion.

##### Justification

- Studies comparing different methods of dissection in NSM were not found in the systematic review, and we are unable to recommend an optimal procedure. It is the consensus of the authors, and consistent with procedures in several of the included studies, that thermal damage should be minimized.

#### 3.4.8. Surgical Factors in NSM: Operative Planning

##### Recommendation

(a) Operative planning should be conducted jointly by the surgeon conducting the mastectomy and the plastic surgeon and include assessment of the blood vessel location and skin perfusion. Perfusion of flaps should be monitored after the operation.

##### Key Evidence

- Several techniques may be used to assess perfusion, including (but not limited to) clinical assessment, MRI, and ICG angiography (e.g., the SPY system). The latter allows for the visualization of blood flow in the tissue of interest and reduced rates of flap loss [120]. It was used in several studies for monitoring of skin flaps and autologous flaps but was not the subject of investigation [121,122,123,124,125,126,127,128,129]. Some studies indicated it was used early but no longer as they became more proficient at clinical assessment [130]. Karin et al. [131] discuss the use of MRI blood flow information to preserve the NAC blood supply.

##### Justification

- The recommendation is based on the consensus of the authors and informed by studies in the systematic review. While studies comparing different methods of assessment in NSM were not found, studies stressed planning and assessment are important to reduce complications including skin and nipple necrosis.

### 3.5. Implant Plane/Location

#### 3.5.1. Background

Early attempts at prepectoral breast reconstruction suffered from unacceptable rates of flap necrosis and capsular contracture [132], as well as a lack of support and implant extrusion. Subpectoral implants were the standard of care for many years, but many patients experienced animation deformity, pain, restricted motion, as well as longer and more complex operations [133,134,135]. The development and use of acellular dermal matrix (ADM), fat grafting, and tissue perfusion assessment technology to assess flap viability have reduced complications and led to more widespread use of prepectoral (and to a lesser extent, dual-plane) reconstruction [132,136]. In the studies included in the systematic review, ADM was usually used with prepectoral implants to provide support of the lower pole and/or to provide an additional layer between the skin envelope and the implant. In partial subpectoral (dual-plane) placement, ADM or other mesh was generally used to cover and support the lower half (lateral pole) of the expander or implant (the portion not under the pectoralis major muscle). Use of ADM is covered in Recommendation 5 and fat grafting in Recommendation 6.

#### 3.5.2. Recommendations

(a) There is a role for both prepectoral and subpectoral implants; risks and benefits will vary, and decisions should be made during consultation between the patient and surgeons.
(b) In patients who are suitable candidates for implant reconstruction and have adequate mastectomy flap thickness and vascularity, prepectoral implants should be considered as they have some advantages over dual-plane or other subpectoral reconstructions.
(c) Patients should be informed of the possibility that subpectoral and submuscular implants may result in long-term animation deformity and related pain and sometimes implant malposition.

#### 3.5.3. Qualifying Statements

- In patients with poor flap quality and vascularization, immediate prepectoral reconstruction was not generally offered; alternatives include subpectoral reconstruction, surgical delay prior to prepectoral reconstruction, or autologous flaps.
- The type and location of implants or autologous tissue should be documented in patient records and available to clinicians conducting follow-up assessments and imaging.

#### 3.5.4. Key Evidence

- Studies found that surgical complications are similar between prepectoral and dual-plane or subpectoral reconstruction [20].
- Animation deformity and associated pain are features of submuscular/subpectoral reconstruction and do not occur with prepectoral reconstruction. Conversion to prepectoral placement in patients with previous subpectoral or dual-plane implants suffering from animation deformity or other implant-related issues was reported in five publications [135,137,138,139,140]. ADM was used in four studies. After revision surgery, all presenting complaints resolved; one study also reported improved shoulder motion and overall breast aesthetics, and one noted improvement in all measures on the BREAST-Q. Minor surgical complications were noted in three studies. Two studies (not in the included studies) found animation deformity in 75% to 100% of patients with subpectoral reconstruction [141,142]. In the study by Becker et al. of 25 patients with subpectoral implants, 80% were bothered and 45% bothered to a significant degree, 48% felt it interfered with daily life, and 28% were having surgical revision. In the study by Nigro et al. of 84 patients with subpectoral dual-plane reconstruction with ADM, 26% considered animation deformity severe and 50% would have preferred a technique to eliminate it.
- Surgical complications may depend more on surgical technique than the plane of the implant. Avila et al. [130] noted evolution of technique to preserve subdermal vascular supply, use of gentle retraction, and careful dissection in the supra-areolar region have improved results. As noted in Question 3, the size of expander/implant and rate of expansion may influence rates of complications. Subpectoral placement was often used if patients were judged not suitable for prepectoral implants, and therefore it is difficult to assign outcomes specifically to the location of implants.

#### 3.5.5. Justification

- It is the consensus of the authors that there is a role for both prepectoral and subpectoral implants. There is strong and consistent evidence that animation deformity and associated pain and other long-term issues may occur with submuscular implants and partial submuscular (dual-plane) implants, and that this may be resolved by replacing implants with prepectoral implants. Short-term post-surgical pain is also lower with prepectoral implants. Surgical complications depend more on patient and surgical factors; evidence comparing these by implant plane/location is weak and insufficient to allow a recommendation as to the preferred plane. As subpectoral implants were often used when patients were judged to be poor candidates for prepectoral reconstruction, no comparative data are available for this subset of patients. Modification to technique, such as indicated in Recommendations 3.3 to 3.7 and the use of ADM (see Recommendation 5) may allow prepectoral reconstruction in patients who would traditionally not be considered candidates.

### 3.6. Use of Acellular Dermal Matrix (ADM) or Synthetic Absorbable Matrix

#### 3.6.1. Background

ADM is usually used with prepectoral implants to provide support for the lower pole and/or to provide an additional layer between the skin envelope and the implant. The expander or implant could be wrapped entirely with ADM (most common), the pocket after SSM/NSM lined entirely with ADM, or ADM used only on the anterior surface and posterior lower pole. The use of ADM has led to being able to perform prepectoral reconstruction safely. In partial subpectoral (dual-plane) placement, ADM or other mesh was generally used to cover and support the lower half (lateral pole) of the expander or implant (the portion not under the pectoralis major muscle). ADM was sutured to the lower border of the pectoralis muscle and in the area of the IMF and is often referred to as a sling providing support to the lower pole of the implant.

#### 3.6.2. Recommendations

(a) Mastectomy flap perfusion should be assessed prior to reconstruction. ADM should not be used in case of poor mastectomy flap perfusion/ischemia that would otherwise be considered unsuitable for prepectoral reconstruction.
(b) Care should be taken in the selection and handling of acellular dermal matrix (ADM) to minimize risks of infection and seroma.
(c) There is insufficient information to recommend a specific human ADM. Sterility level may be a factor in the selection of a product.
(d) Undue tension on the mastectomy flaps should be avoided.
(e) Absorbable synthetic mesh may be an alternative to human ADM; however, comparative information is very limited, and no recommendation can be made.

#### 3.6.3. Qualifying Statements

- Few studies with a direct comparison of reconstruction in the same plane with and without ADM were included in the systematic review. Most studies compare prepectoral reconstruction with ADM to subpectoral reconstruction (with or without ADM).
- Limited data from small studies suggest that prepectoral reconstruction without ADM may be feasible in some patients and has similar complications with and without ADM [20].
- Dual-plane reconstruction without ADM appears more common than prepectoral reconstruction without ADM. Alternatives to the use of ADM in dual-plane reconstruction exist, including an inferolateral incision instead of IMF incision to provide more support, using fascia of serratus anterior muscle, or using the mastectomy skin alone. Repair of the IMF area may be required.
- ADM use has been associated with an increased risk of infection and seroma. Risks may vary with the type and preparation of ADM; seroma rates are observed to be lower when ADM is perforated or meshed.
- Fenestration generally refers to the process of creating slits (as performed in meshing) but sometimes refers to perforations and this term is therefore ambiguous. In several studies, adding perforations or meshing was performed by the surgeon immediately prior to placement. These treatments are now available commercially as well but at added cost.
- Bioabsorbable mesh has been used in several studies and may be beneficial, but the information is insufficient to rank any compared with the commonly used human ADM or each other.

#### 3.6.4. Key Evidence

- Six studies compared AlloDerm^TM^ (LifeCell Corporation, Branchburg, NJ, USA) to FlexHD^®^ (Musculoskeletal Transplant Foundation [MTF], Edison, NJ, USA) [20]. No consistent differences were found.
- AlloDerm (aseptic/freeze-dried) had a higher risk of infections than AlloDerm RTU (ready-to-use, sterile) [20]. AlloDerm (aseptic/freeze-dried) was replaced in 2010 by AlloDerm RTU which is terminally sterilized to a Sterility Assurance Level (SAL) of 10^−3^ [143].
- Palaia et al. [144] found fenestrated ADM had a lower risk of seroma (11.1% vs. 20.0%, *p* = 0.0098). A laboratory study of biomechanical properties of meshed and perforated ADM [145] found the meshed pattern increased surface area by 97.5%, while perforations increased surface area by 0.30% and 0.59%. Egress rate of fluid was 1.974 s with meshing, and 6.504 and 10.369 s with two patterns of perforation.
- Four studies had comparisons of synthetic mesh [146,147,148,149]. Data comparing ADM, poly-4-hydroxybutyrate (P4HB; GalaFLEX^TM^ [Becton, Dickinson and Company, Lexington, MA, USA] or Phasic^TM^ [Becton, Dickinson and Company, Franklin Lakes, NJ, USA]), and TIGR^®^ Matrix (Novus Scientific, Uppsala, Sweden) were found but are insufficient to make any recommendations.
- Warren Peled et al. [58] conducted immediate expander-implant reconstruction after TSSM. The inferior aspect of the pectoralis major was left intact at its inferior origin superior to the IMF. ADM was sutured laterally and inferiorly to the chest wall at the inferolateral aspect of the expander. When ADM was not used the expander was placed in the same pocket but without the added coverage. The group with ADM had less infection, unplanned return to the operating room, and expander-implant loss. The benefit was greater in patients with thin flaps and those receiving RT and it was suggested ADM may not be needed in patients with lower risk. The no ADM group had more risk factors (more therapeutic cases; more RT, chemotherapy, and axillary lymph node dissection) that were not adjusted for.
- Nahabedian et al. [150] compared dual-plane reconstruction with or without ADM and found no differences in infection rates. Other studies [151,152,153] found dual-plane reconstruction with ADM had more complications than dual-plane or submuscular without ADM.

#### 3.6.5. Justification

- As indicated in Recommendation 4, there are some advantages to the use of prepectoral plane and dual-plane implants. Prior to the development of ADM for this purpose, prepectoral implants were rarely used. The biggest barriers to ADM use are cost and availability. Most studies with prepectoral and dual-plane reconstructions used ADM, and ADM appears to allow this without an increase in major complications. Infection and seroma are two of the complications that are sometimes reported to be higher with ADM use, and care should be taken to minimize these, including product selection, form/preparation, and handling of ADM.
- AlloDerm (aseptic/freeze-dried) was the most commonly studied ADM but is no longer in use as it was replaced in 2010 by AlloDerm RTU. Data comparing different ADM products that are relevant to current practice are limited. Complication rates were similar, and information is insufficient to recommend a specific product.
- Some surgical groups have conducted prepectoral or dual-plane reconstruction with good results without ADM. These reports are limited and may require adaptation of surgical techniques or be applicable to only a subset of patients. The authors recommend continued use of ADM as this increases the reconstructive options and reduces some long-term complications of submuscular implants. We acknowledge that technical improvements in mastectomy and reconstruction may reduce the use of ADM.

### 3.7. Autologous Fat Grafting (Lipofilling)

#### 3.7.1. Recommendations

(a) Fat grafting is recommended as a treatment for contour irregularities.
(b) Fat grafting is recommended as a treatment for rippling following implant-based reconstruction.
(c) Fat grafting may be used to improve tissue quality of the mastectomy flap after radiotherapy (RT).
(d) Patients undergoing radiologic exams should indicate that they have undergone reconstruction including autologous fat transfer.
(e) Evidence on total fat grafting is more limited, and a recommendation cannot be made at this time.

#### 3.7.2. Qualifying Statements

- Outcomes are highly dependent on the method of fat harvesting and treatment, and on the amount and location of injection. Excess pressure due to overfilling can cause fat necrosis and lower rates of fat survival.
- Palpable masses as a result of fat necrosis may occur in patients who have received fat transfer. These are generally benign on imaging and can be identified without biopsy in most cases.
- Enrichment/enhancement of stem cells is an area of active research but was not within the scope of this work.
- The optimal timing of fat grafting is unclear and may vary according to indication. The first session of fat grafting is usually at the time of expander-implant exchange or as a revisionary procedure several months after the final implant or autologous reconstruction, although there are sometimes reasons to use it at the time of expander insertion or autologous flap placement. In patients with poor mastectomy skin flap quality, fat grafting prior to expander insertion (for delayed-immediate reconstruction) or expander-implant exchange (in case or radiation damage) may improve tissue quality and reduce complications. In patients requiring RT, fat grafting often occurs 2 to 6 months after the end of RT.

#### 3.7.3. Key Evidence

- The systematic review found no statistically significant differences in local or locoregional recurrence, distant metastasis, OS, DFS, or locoregional recurrence-free survival for patients with or without autologous fat grafting [20].
- The MROC study [154] included 165 patients with contour irregularities or volume deficits and fat grafting between years 1 and 2. Patients with fat grafting had lower QoL than controls before fat grafting and QoL similar to controls afterwards, suggesting that fat grafting is beneficial in patients with contour irregularities or other defects.
- Calabrese et al. [155] reported lower rates of capsular contracture (7.14% vs. 21.53%) with fat grafting, as well as lower rates of hematoma, implant displacement/rotation, pain, and revision surgery within 3 years. Using the BREAST-Q, patients with fat grafting reported significantly better for several scales of Satisfaction (softness, natural appearance and feel to touch, natural part of body) and of Physical Well-Being (pain in chest muscles; breast tightness, pulling, nagging feeling, sharp pains, aching feeling, throbbing feeling).
- A randomized controlled trial (RCT) by Gentilucci et al. [156] evaluated the effectiveness of fat grafting after RT but prior to expander-implant exchange compared with a group without fat grafting. Skin biopsies evaluated adipose thickness and found a significant increase with lipofilling (178% after the third fat injection). There was a qualitative and quantitative improvement of tissues with lipofilling. The study had a 1-year follow-up and concluded fat grafting reduced PMRT damage and improved QoL and reconstructive surgery outcomes.
- Calabrese et al. [155] reported less pain in the group with fat grafting. Other comparative studies on relief of pain and postmastectomy pain syndrome meeting our criteria were not included in our systematic review but this was mentioned in smaller studies [157,158,159,160], as well as separate systematic reviews on the topic [161,162].
- The Breast Trial (NCT02339779) [163,164,165,166] compared autologous fat transfer alone in conjunction with external expansion to implants. Primary outcomes were QoL as measured by the BREAST-Q, with other outcomes of safety (complications or adverse events), aesthetic evaluation, and sensibility. Breast-Q scores and QoL including Satisfaction with Breasts (70.3 vs. 60.4, *p* = 0.002), Physical Well-Being Chest (79.9 vs. 72.3, *p* = 0.007), and Satisfaction with Outcome (73.9 vs. 66.3, *p* = 0.04) were better in the fat transfer group. There were no differences in oncologic events up to 1 year; long-term follow-up is ongoing. Exploratory analysis found sensibility in a subset of patients measured at least 1 year after final surgery was significantly better in the fat transfer group.
- Cason et al. [167] reported on imaging and biopsies after fat grafting. They found more palpable masses (38.0% vs. 18.3%). These were mostly normal or benign on imagining and biopsies were only required in 11.8% vs. 7.5% of patients. Fat necrosis was the most frequent radiologic interpretation and is generally identifiable on imaging. Several other studies not included in our review also reached similar conclusions [168,169,170]. Fracol et al. found 8.6% of breasts developed palpable nodules; these were identified by ultrasound or mammography as presumed fat necrosis (38.2%), benign lesions (27.6%), presumed oil cysts (17.1%), indeterminate (8.9%), and concerning for malignancy (8.1%). Fiaschetti et al. [169] indicated that experienced radiologists can distinguish lipofilling changes from malignant alterations.
- The UK guideline on lipofilling of the breast [171] provides guidance on indications, training, and techniques. Applications in post-mastectomy reconstruction include its use together with implants or autologous flaps in primary reconstruction, total breast reconstruction (best suited to small-breasted women), and improving the quality of irradiated tissue before or during implant-based reconstruction.

#### 3.7.4. Justification

- There is strong evidence that fat grafting is an oncologically safe procedure and improves PROs and QoL. The use of fat grafting for improvement in contour irregularities and reduction in rippling is well established. Based on this, its continued use is recommended. Training and technique are important for optimal outcomes, including the appearance and safety of the breast and site of liposuction, and minimizing fat necrosis. Some studies reported the benefit of fat grafting to relieve postmastectomy pain syndrome; however, the authors believe there is not enough evidence at this time to make a recommendation. While several studies, including the BREAST Trial with external expansion and others with internal or no pre-expansion have used fat grafting alone (without implant or autologous flaps) with good results, this is not common practice in Ontario and authors believe expertise is not currently available. From a patient perspective, total reconstruction with fat grafting should be developed and offered as an alternative to implants and autologous flaps. This would provide an additional option for those averse to implant reconstruction and who are not candidates for autologous flap reconstruction.

## 4. Discussion

As indicated in the accompanying systematic review [20,21], reconstruction may improve the quality of life of women who have a mastectomy to treat breast cancer. We concluded that NSM (compared to SSM) does not increase rates of recurrence or reduce survival provided the nipple is not involved and therefore recommend that NSM should be considered in patients who are candidates for SSM if there is no clinical and pathological evidence of nipple involvement. We also concluded that autologous fat grafting does not increase rates of subsequent oncologic events and therefore recommend its use as a treatment for contour irregularities, rippling after implant-based reconstruction, and to improve tissue quality prior to reconstruction or expander-implant exchange (especially after RT). Evidence is promising but considered insufficient to make a recommendation regarding total fat grafting (i.e., reconstruction of a breast mound with fat grafting without implants or autologous flaps).

Reconstruction results vary from very poor to excellent appearance, with a strong dependence on training and skill of both the surgeon conducting the mastectomy and the surgeon performing reconstruction. Preoperative planning and collaboration of the surgeons including a decision to use SSM, ASM, or NSM, and determining the location of incisions is recommended. This may reduce necrosis by preserving blood flow and optimize retention or restoration of at least partial sensation. Many of the decisions regarding reconstruction depend on patient and disease characteristics, as well as patient preferences. Because of this, much of the literature reported outcomes for two or more approaches in groups of patients that were not equivalent Studies comparing different approaches in patients with equivalent characteristics (or with adjustment by propensity score matching or multivariate analysis) were limited and often insufficient for us to reach conclusions except of the type “In patients considered candidates for both approaches, both should be considered. Complication profiles may vary.” In SSM and NSM, skin flap thickness and perfusion are key factors that may influence whether a patient is a candidate for prepectoral or immediate reconstruction. Comorbidities and habits such as smoking may influence perfusion and thus rates of complications and aesthetic outcomes and therefore considered along with other factors and preoperative perfusion assessment/mapping. In patients considered suitable candidates for both prepectoral and subpectoral implants, prepectoral implants do not result in animation deformity and associated pain and may therefore be preferred. Sufficient support of the implant (e.g., with ADM, choice of incision to reduce IMF disturbance, selection of appropriate implant size) is required.

Traditionally, mastectomy results in total loss of sensation. Reconstruction is often limited to restoring visual/aesthetic results, which can be excellent in some patients. Lack of feeling can result in unawareness of burns, not feeling physical contact such as hugs, and inability to feel touch or experience sexual arousal. Studies have indicated retention or restoration of partial sensation is possible in some patients. This may involve connecting nerves in autologous flaps to those in the reconstruction site, minimizing damage to nerves in NSM or SSM by careful planning of incisions and avoiding thermal damage, or nerve repair by coaptation or with nerve grafts (allograft or autograft). Nerve allografts are expensive, and costs are not covered by the provincial health insurance in Ontario. Nerve repair is not standard of practice in Ontario for breast reconstruction and sufficient expertise is not available for this to be used routinely. Further research and incorporation of these techniques in surgical training may lead to more widespread use in the future.

### 4.1. Other Limitations

During internal and external review of this guideline prior to its completion, it was noted that mastectomy is conducted by surgeons of varying degrees of specialization and includes general surgeons (especially at smaller hospitals) and surgical oncologists. Some surgeons do not have proficiency in conducting NSM or SSM and these may not be offered. Preoperative planning and incision locations may not be optimal for subsequent reconstruction. Plastic surgeons may not be involved in the initial decisions or mastectomy planning, or availability may be a determining factor in the selection between immediate and delayed reconstruction. In Ontario, access to and resources for breast reconstruction vary across the province. These are considered to be healthcare system issues and outside the scope of this work but important for implementation.

Plastic/reconstructive surgeons have varying levels of expertise and all will not be able to offer both implant and autologous reconstruction. Surgical training is also an issue for incorporating the use of autologous fat grafting. Results in the literature vary widely, and this appears due to the degree of training and expertise of the plastic/reconstructive surgeon.

The systematic review on which this guideline is based included primarily retrospective studies that may have additional confounding factors for which adjustment was not made. Data was limited for many of the comparisons of interest. Surgical complications are influenced by patient characteristics and the reconstructive approach may need to be adjusted on an individual basis. Cost analysis was outside the scope of this work.

### 4.2. Review and Update

The currency of each document is ensured through periodic review and evaluation of the scientific literature and, where appropriate, the addition of newer literature to the original evidence. This is described in the PEBC Document Assessment and Review Protocol [172]. For the full guideline and systematic review and subsequent updates, please visit the OH (CCO) website at https://www.cancercareontario.ca/en/guidelines-advice/.

## Data Availability

This work is based on data from publicly available and referenced sources. Data sharing is not applicable to this article.
